# Rear-Sided Passivation by SiN_x_:H Dielectric Layer for Improved Si/PEDOT:PSS Hybrid Heterojunction Solar Cells

**DOI:** 10.1186/s11671-016-1505-7

**Published:** 2016-06-28

**Authors:** Yiling Sun, Pingqi Gao, Jian He, Suqiong Zhou, Zhiqin Ying, Xi Yang, Yong Xiang, Jichun Ye

**Affiliations:** School of Energy Science and Engineering, University of Electronic Science and Technology of China, Chengdu, 611731 People’s Republic of China; Ningbo Institute of Materials Technology and Engineering, Chinese Academy of Sciences, Ningbo, 315201 People’s Republic of China

**Keywords:** Si/PEDOT:PSS, Hybrid solar cells, SiN_x_:H passivation, Photolithography

## Abstract

Silicon/organic hybrid solar cells have recently attracted great attention because they combine the advantages of silicon (Si) and the organic cells. In this study, we added a patterned passivation layer of silicon nitride (SiN_x_:H) onto the rear surface of the Si substrate in a Si/poly(3,4-ethylenedioxythiophene):poly(styrenesulfonate) (PEDOT:PSS) hybrid solar cell, enabling an improvement of 0.6 % in the power conversion efficiency (PCE). The addition of the SiN_x_:H layer boosted the open circuit voltage (*V*_oc_) from 0.523 to 0.557 V, suggesting the well-passivation property of the patterned SiN_x_:H thin layer that was created by plasma-enhanced chemical vapor deposition and lithography processes. The passivation properties that stemmed from front PEDOT:PSS, rear-SiN_x_:H, front PEDOT:PSS/rear-SiN_x_:H, etc. are thoroughly investigated, in consideration of the process-related variations.

## Background

Over the past several decades, crystalline silicon (c-Si) solar cells have dominated the commercial solar cell market due to multiple factors, such as high power conversion efficiency (PCE) [1], abundance of raw materials, free of toxicological issues, and well-established processing techniques. However, this type of solar cells suffers from drawbacks such expensive processing and large material consumption due to high-temperature treatment and thick substrate required. In recent years, organic photovoltaics emerge as a promising technology in the solar energy field, thanks to simple processing and low material consumption [2–4]. The development of organic solar cells is faced by a grand challenge: the PCE is relatively low due to the low electron–hole separation efficiency. The emergence of c-Si/organic hybrid photovoltaics offers a possible route to low-cost and high-efficiency solar cells by combining the advantages of c-Si and organic materials [5–7]. Recently, poly(3,4ethylenedioxythiophene)/poly (styrenesulfonate) (PEDOT:PSS) has stimulated intense interest in the research community because of its advantageous properties with respect to light transmission and hole conductivity. Up to now, the PCE of PEDOT:PSS hybrid solar cells has been improved to above 13 % [8–10] as a result of efforts in several areas including interface modification [11, 12], surface texturing on Si [13–17], and property tuning of PEDOT:PSS [18]. Typical improvements related to the rear side is to add an ultra-thin interfacial layer of LiF [19], LiQ [9], or CsCO_3_ [20] between the c-Si layer and the back electrode, with the aims to reduce contact resistance and enhance the rear electric field. With this design, the short circuit current density (*J*_sc_) and open circuit voltage (*V*_oc_) are both enhanced. However, it is critical to precisely control the thickness of these kinds of layers at a certain value, in order to achieve a satisfied contact resistance while not hindering charge carrier collection.

For the purpose of passivating the n-type c-Si, hydrogenated silicon nitride (SiN_x_:H) is an ideal candidate material. SiN_x_:H [21], conventionally deposited by plasma-enhanced chemical vapor deposition (PECVD), is known to be widely used in the Si-based solar cell processing. This dielectric layer contains considerable amount of hydrogen bonds and positive charges (typically several 10^12^ cm^−2^) [22], offering good chemical and field-effect passivation on H-terminated n-type emitter [23]. To date, surface recombination velocities (*S*_eff_) below 10 cm/s have been achieved though PECVD method [22, 24].

In this study, we fabricated a hybrid c-Si/organic solar cell with an added passivation layer of PECVD-SiN_x_:H at the rear side and investigated its characteristics. First, the PECVD-SiN_x_:H layer was thoroughly characterized by surface recombination velocity, focusing on its relations to some aspects including thickness and chemical bond. Second, photoresist was served to protect the SiN_x_ layer, and chemical etching with diluted hydrofluoric acid (HF) was used to obtain a partial passivation layer with a SiN_x_-to-substrate ratio of 60 %. After that, a PEDOT:PSS film was formed on the front side of the substrate by spin-coating, followed by the formation of grid-Ag/full-Al contact layers on the front and rear sides by thermal evaporation. A comparison of the SiN_x_:H-passivated device and control sample showed perceivable increases in both *J*_sc_ and *V*_oc_, improving the PCE by 0.6 to 9.0 % under the simulated solar illumination (AM 1.5, 100 mW/cm^2^).

## Methods

A Si wafer (n-type, single-side polished, float zone, 20 × 20 mm, 300 ± 15 μm in thickness, resistance 3-5 Ωcm) underwent standard RCA (Radio Corporation of American) [25] cleaning and 8 % (volume ratio) HF cleaning. Then, a SiN_x_:H passivation layer was deposited upon the double sides of the Si substrate from the gas mixture of SiH_4_ (5 sccm), NH_3_ (40 sccm), and Ar (40 sccm) for 10 min at a temperature of 350 °C with a pressure of 70 Pa. The prepared films have a thickness of about 100 nm that was measured with a scanning electron microscope (SEM).

A layer of negative photoresist was coated on the SiN_x_:H layer by spin-coating at a speed of 3000 rpm for 30 s. Then, the masked Si/SiN_x_:H layer was exposed in the UV for 90 s. The exposed portion of the negative photoresist was then washed away using a developer, and the surface underwent an etching process in 0.25 % HF solution for 30 s, removing the portion of the SiN_x_:H layer without the protection of photoresist. After washing away the remained photoresist by acetone, a Si substrate partially covered by a SiN_x_ film was obtained.

**Fig. 1:**
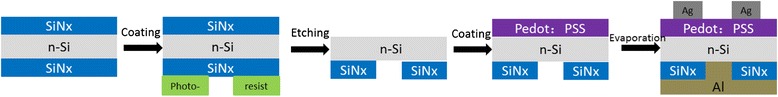
Process flow of the fabrication of Si/PEDOT:PSS hybrid solar cell with a patterned SiN_x_:H passivation layer

Next, the front side of the sample was spin-coated with PEDOT:PSS solution at a speed of 3000 rpm for 1 min, and then heated on the on a hotplate at 130 °C for 10 min to remove the solvents. Finally, a 150-nm-thick grid Ag layer and a 200-nm-thick Al layer were thermal-evaporated on the front side and rear side of the sample, respectively. The fabrication processes of the Si/PEDOT:PSS hybrid solar cell with a patterned SiN_x_:H passivation layer was schematically shown in Fig. 1.

The surface topography and thickness of the patterned SiN_x_:H passivation layer were observed by SEM (Hitachi S-4800 SEM). The chemical bonding characteristics of the SiN_x_:H layers were obtained by attenuated total reflectance Fourier transform infrared spectroscopy (ATR-FTIR, Harrick) and X-ray photoelectron spectroscopy (XPS, AXIS Ultra DLD). Using a microwave photoconductance decay (μ-PCD) technique (WT2000PVN, Semilab), the minority carrier lifetimes of the SiN_x_:H layers were characterized. After calibrating the irradiation intensity of the standard silicon photovoltaic device (Oriel, model 91150 V), the current density–voltage (*J–V*) characteristics of the hybrid solar cells were tested with a Keithley 2400 digital source meter (Keithley) under simulated sunlight (100 mW/cm^2^) illumination provided by a xenon lamp (Oriel) with an AM 1.5 filter. The open area of the cells was 0.7 cm × 0.8 cm with 0.11 cm^2^ area shaded by the grid of Ag electrodes. Newport silicon detector and 300-W xenon light source with a spot size of 1 × 3 mm was used to measure the external quantum efficiency (EQE).

## Results and Discussion

### SiN_x_:H Surface Topography

**Fig. 2:**
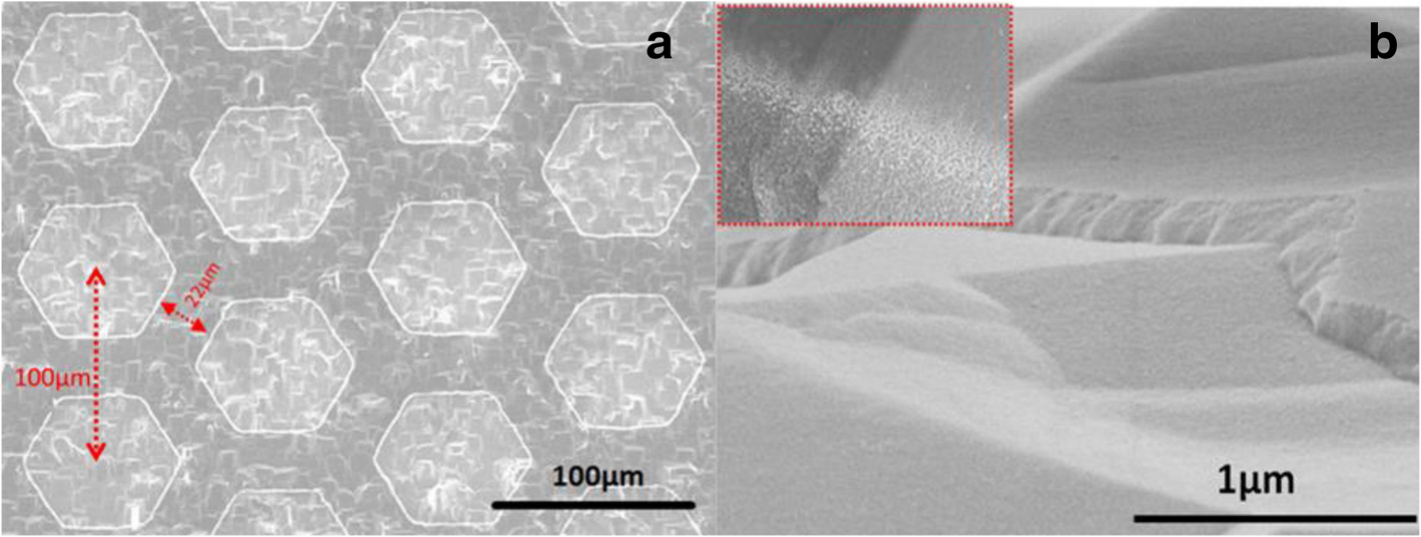
SEM images of the passivation layer. **a** is the top view. The hexagons are SiN_x_ patterns, with a center-to-center distance of 100 μm and a spacing of 22 μm. **b** represents SiN_x_ film with a good etching result and uniform coverage, without residual photoresist and symptom of overetching. The *inset* shows the pores as a result of HF horizontal etching.

The SiN_x_:H was finally patterned into many hexagons, with a center-to-center distance between adjacent hexagons of 100 μm and minimum spacing between hexagons of 22 μm. Through this design, we obtained passivation patterns with a passivation-to-substrate coverage ratio of 60 %. Figure 2a shows the patterns after HF etching (0.25 %, 30 s) and ultrasonic cleaning with acetone, where the unpolished Si surface was covered by a uniform layer of hexagonal SiN_x_:H. The HF etching (0.25 %, 30 s) produced a steep SiN_x_:H edge without residual photoresist or a symptom of overetching (Fig. 2b). It was observed that during the etching process, excessively high concentration of HF or extra etching time could result in pore-like configuration on the SiN_x_:H (Fig. 2 add-on). This is because the HF penetrated into the interface between photoresist and SiN_x_ and horizontal overetching occurred. Apart from creating pores, horizontal overetching could also bring negative impact on or even neutralize the passivation. It was observed that performing etching using HF with a concentration of 0.25 % for 30 s produced optimal passivation and the micron-scale channels between the hexagons could offer enough depth to width ratio for thermal-evaporating electrode materials.

### Passivation of the SiN_x_:H Layer

**Fig. 3:**
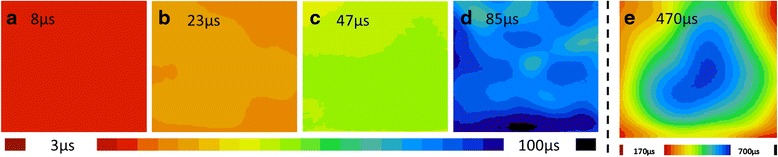
The mapping of minority carrier lifetime for the Si substrates without passivation (**a**), with coating of front PEDOT:PSS (**b**), with coating of patterned SiN_x_:H at the rear side only (**c**), with coating of PEDOT:PSS at the front and patterned SiN_x_:H at the rear (**d**), with coating of double-sided SiN_x_:H in full area (**e**), respectively. The average lifetime was indicated in each mapping images

The minority carrier lifetime depends on the recombination on the surface and inside of the bulk, as expressed in Eq. 1.

1 $$ \frac{1}{\tau_m}=\frac{1}{\tau_b}+\frac{2{S}_{\mathrm{eff}}}{W} $$

where *τ*_m_ is the measured lifetime, *τ*_b_ is the bulk carrier lifetime, *S*_eff_ is the surface recombination velocity, and *W* is the wafer thickness. The minority carrier lifetime of Si wafers with different surface treatments was mapped and shown in Fig. 3. The average carrier lifetime increased to 470 μs after the deposition of a 100-nm SiN_x_:H layer, in comparison to 7 μs that was measured in the reference of a 300-μm-thick raw FZ-Si wafer (Fig. 3a). Assume that *τ*_b_ is infinite in comparison with *τ*_m_, and *S*_eff_ can be calculated as low as 30 cm/s using Eq. 1. Unfortunately, the HF etching process via opening on the SiN_x_:H layer will cause a dramatic reduction in the lifetime of the minority carriers, i.e., from 470 to 47 μs (Fig. 3c). This figure is only 10 % of that of the fully coated sample with SiN_x_:H, but still about seven times of that of the reference sample without any passivation (Fig. 3a). To further study the impact of passivation properties both from the front PEDOT:PSS and rear SiN_x_:H layer, the patterned sample and the reference sample were spin-coated with a PEDOT:PSS film on the top surface. The comparison revealed that the average lifetime of the minority carrier of the sample solely coated by PEDOT:PSS was 23 μs (Fig. 3b), while the one of the sample with both PEDOT:PSS and patterned SiN_x_:H was 85 μs (Fig. 3d). Inferred from this improvement in carrier lifetime, an implied increase of 0.020 V in *V*_oc_ and 1 % in PCE are both expected.

### Chemical Bond Structure of the SiN_x_:H Layer

**Fig. 4:**
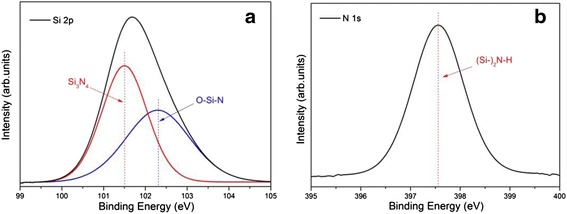
The Photoemission (XPS) spectra for (**a**) Si 2p and (**b**) N 1s of the SiN_x_:H film

XPS and FTIR were used to confirm the chemical bond characteristics of the SiN_x_:H film. Figure 4 shows the XPS spectra of the SiN_x_:H film, with all binding energy values being calibrated to the contaminant carbon peak at 284.8 eV. XPS spectrum indicates that the Si/N molar ratio is 40:29. The peak of Si 2p spectra was located around 102 eV (Fig. 4a), composed of the Si 2p signal of the Si_3_N_4_ group at 101.5 eV [26] and the signal of O–Si–N group at 102.3 eV [27]. There exists an oxygen element in the SiN_x_:H film, that is because the sample had adsorbed oxygen or H_2_O in the air. Figure 4b shows that N 1s peak consists of a single symmetric peak at 397.6 eV. According to an earlier work by Shallenberger et al. [28], the peak located at 397.6 eV is most likely related to a specific bonding style (Si–)_2_N–H i.e., one N atom is bonded to two Si atoms and one H atom. Meanwhile, no other N 1s peaks like (Si–)_2_N–O at 399.7 eV, Si–N(–O)_2_ at 402.8 eV, and NO_3_– at 407 eV were found in the spectrum. Therefore, the impact of the absorbed water molecules is excluded and Si–N and Si–Si–N bonds are most likely the dominant bonding types in the passivation film, which will contribute mainly to the field-effect passivation.

**Fig. 5:**
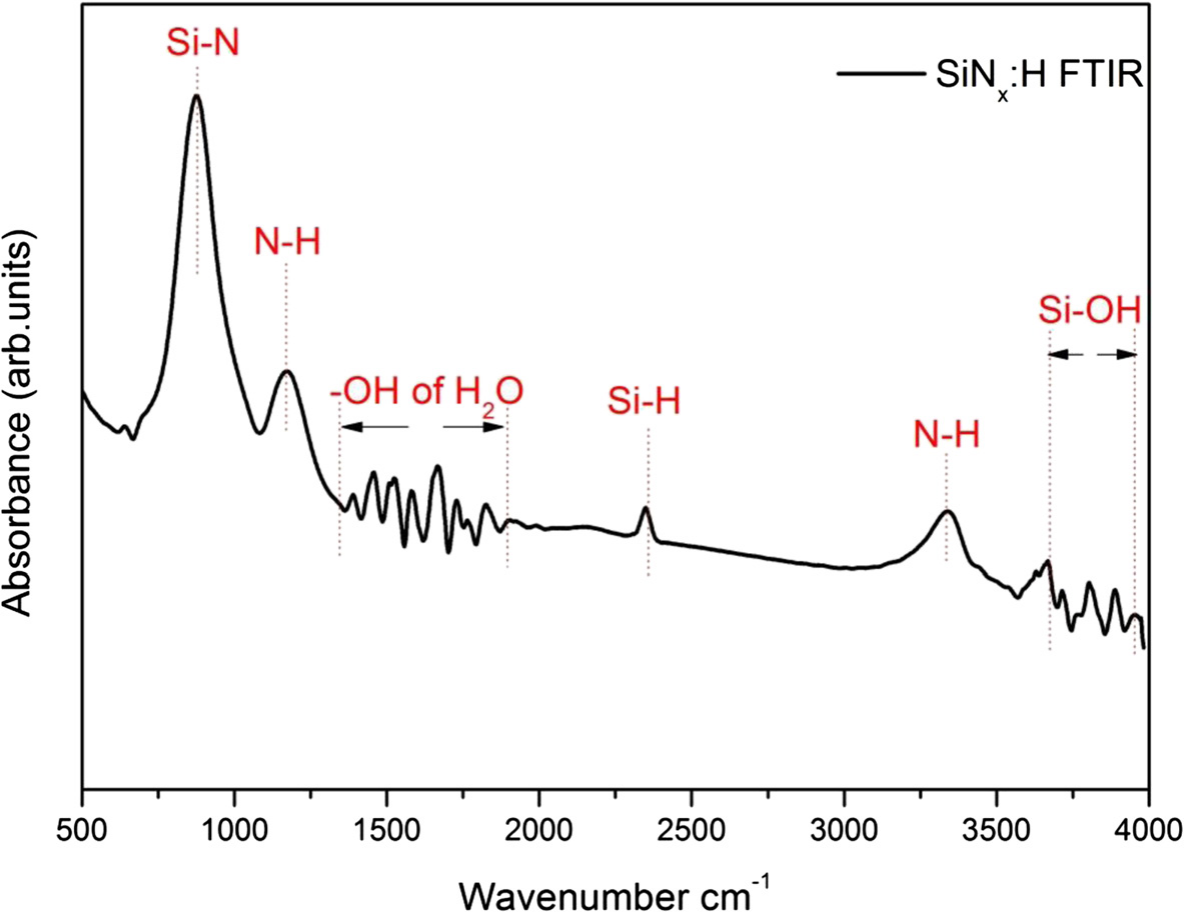
ATR-FTIR curve of SiN_x_:H film

ATR-FTIR analysis was carried out in order to obtain details on the chemical state of the SiN_x_:H film. ATR-FTIR absorption curves in Fig. 5 show a dominant absorption feature around 878 cm^−1^ which can be ascribed to the Si–N bending stretching vibration [29]. According to earlier ATR-FTIR analyses reported by Patil et al., the peak around 2349 cm^−1^ occurring in the ATR-FTIR spectrum resulted from the vibration of Si–H bond [30]. The N–H absorption bond at the 1173 and 3340 cm^−1^ are typically ascribed to the SiN_x_:H film [31, 32]. In addition, the peaks in the region from 3580 to 3670 cm^−1^ and around 1650 cm^−1^ peaks can be explained by the absorption of the H_2_O molecules on the surface of the SiN_x_:H film [33]. The above analysis on the chemical bond structure shows good agreement with the analysis by XPS, indicating that Si–N bond is the dominant bond type in the SiN_x_:H film. What is more, hydrogen-related bonds such as N–H and Si–H were observed in ATR-FTIR spectrum, which may offer good chemical passivation on H-terminated n-type Si.

### Photovoltaic Characteristics of the Solar Cells

**Fig. 6:**
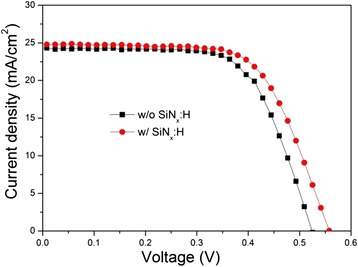
Current–voltage curves of Si/PEDOT:PSS devices with or without a passivation layer of patterned SiN_x_:H

To verify the effect of the patterned passivation layer at the rear side, we fabricated a few heterojunction solar cells with/without a SiN_x_:H layer. Figure 6 shows the current density–voltage (*J–V*) characteristics of the related Si/PEDOT:PSS hybrid solar cells. Table 1 lists the average values of *J*_sc_, *V*_oc_, fill factor (FF), and PCE of the fabricated devices. The passivation effect of the SiN_x_:H layer on the rear surface is directly reflected in the measured value of *V*_oc_ of the Si /PEDOT:PSS solar cell. The *V*_oc_ of the SiN_x_:H layer coated samples increased by 0.033 V in comparison with the samples without a passivation layer, reaching a maximum of 0.557 V. This can be attributed to the decrease of the charge carrier recombination that occurred on the rear surface. The little bit increase in *J*_sc_ (from 24.0 to 24.8 mA/cm^2^), possibly because of the enhanced capability of carriers collection at a longer wavelength range. The series resistance (*R*_s_) reached an acceptable value of 10.39 Ωcm^2^ for the SiN_x_:H-coated hybrid cells which is comparable to the *R*_s_ of 7.95 Ωcm^2^ for the reference sample. It is reasonable for the little bit increase in *R*_s_ because of the reduced contact area between the Si and the rear electrodes. Similarly, a little degradation in FF was also observed. For the above reasons, the addition of the SiN_x_:H layer did not bring significant changes to the PCE of the SiN_x_:H-coated solar cells, with a PCE of 9.0 % and only 0.6 % higher than the reference sample. Better results can be expected if more efforts are made to optimize the coverage percentage and the contact properties of the rear side.

**Table 1:** Photovoltaic characteristics of Si/PEDOT:PSS heterojunction device with or without a SiN_x_:H layer

	*V*_oc_ (V)	*J*_sc_ (mA/cm^2^)	FF (%)	PCE (%)	*R*_s_ (Ωcm^2^)	Rsh (Ωcm^2^)
W/O SiN_x_:H	0.523 ± 0.011	24.0 ± 0.18	67.78 ± 0.27	8.40 ± 0.21	7.95 ± 0.42	2570.80 ± 5.78
W/ SiN_x_:H	0.557 ± 0.014	24.8 ± 0.22	65.24 ± 0.22	9.02 ± 0.15	10.39 ± 0.40	5544.18 ± 4.69

Note: Values were obtained by averaging five devices with a calculated confidence interval of 95 %

**Fig. 7:**
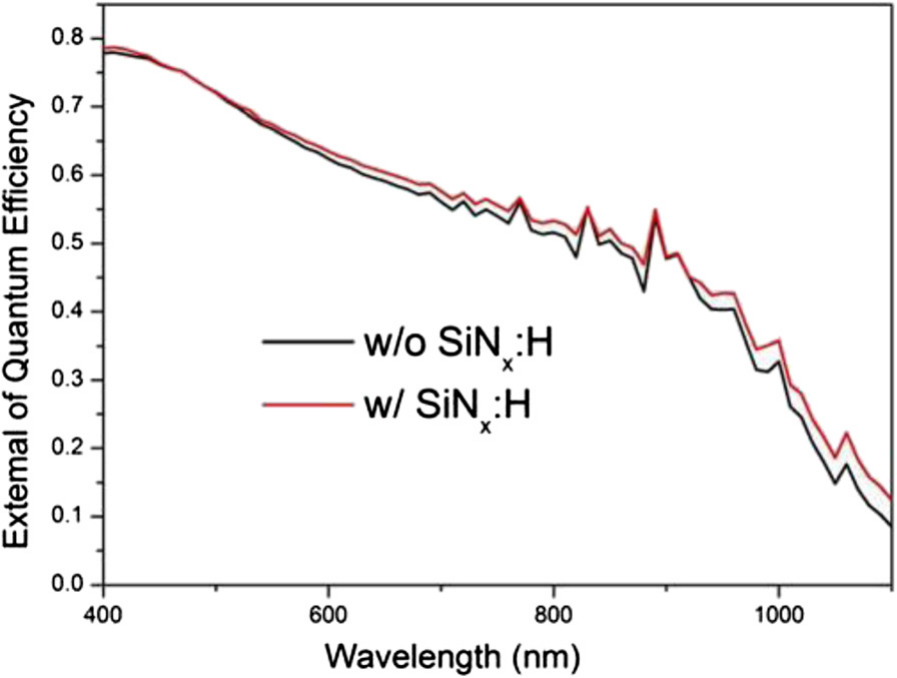
EQE curve of Si/PEDOT:PSS cells with or without a SiN_x_:H passivation layer

The EQE was measured, as shown in Fig. 7. Cells with the SiN_x_:H layers displayed a higher EQE value in the visible and near-infrared region in comparison with the reference cells, which is consistent with the increase of *J*_sc_, benefiting from recombination suppression occurring on the rear surface.

**Fig. 8:**
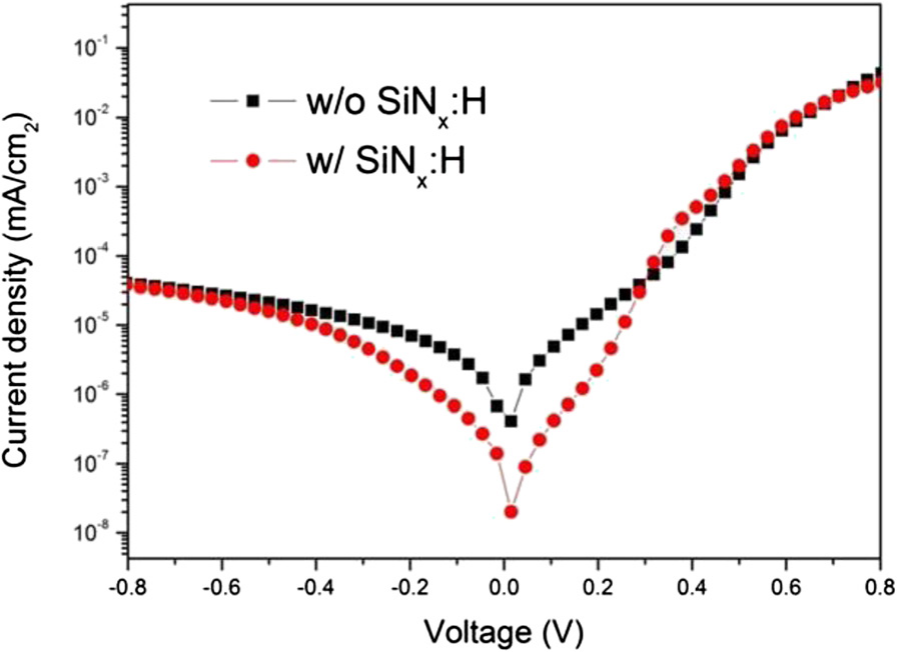
Dark *J–V* curve of Si/PEDOT:PSS devices with or without SiN_x_:H passivation layer

In order to further investigate the performance of the cells with the SiN_x_:H passivation layer, the dark *J*–*V* characteristics were measured and the results were plotted in Fig. 8. It was observed that saturation current density (*J*_s_) reduced significantly after the addition of the SiN_x_:H layer on the rear layer. The typical rectifying characteristic curve indicated that SiN_x_:H-coated hybrid cells exhibited better behaviors characteristic of heterojunction as well as a diode. The dark *J–V* curves were simulated according to the thermionic emission model as follows:

2 $$ J={J}_{\mathrm{s}}\left[ \exp \left(\frac{eV}{nkT}\right)-1\right] $$

3 $$ {J}_{\mathrm{s}}={A}^{\ast }A{T}^2 \exp \left(-\frac{\varPhi_{\mathrm{bi}}}{KT}\right) $$

where *J*_s_ is the reverse saturation current density value, *V* is the applied voltage, *T* is the absolute temperature (298 K), *k* is the Boltzmann constant (1.38 × 1023 m^2^ kg s^−2^K^−1^), *q* is the electronic charge (1.6 × 10^−19^C), *A* is the contact area, A* is the effective Richardson constant (about 252 A cm^−2^ K^−2^ for n-type Si), and *Φ*_bi_ is the barrier height of Schottky diode. From the dark *J–V* curves under the forward bias condition (Fig. 8) and Eqs. 2 and 3, the *n*, *J*_s_, and *Φ*_bi_ of the heterojunction solar cells were extracted, as listed in Table 2. The device with the SiN_x_:H layers displayed a *J*_s_ value of 5.55 × 10^−7^A/cm^2^, approximately a half of that of the cells without the SiN_x_:H layer (which was 1.08 × 10^−6^A/cm^2^). Here, the diode ideality factor *n* in this Schottky diode is linked to the quality of the p–n junction which is influenced by the recombination velocity on the rear surface of Si. The SiN_x_:H-coated device has a smaller *n* value of 2.45, which implied that lower density of the defect centers in the space charge region due to the better passivation quality of the SiN_x_:H. And the *Φ*_bi_ value was also increased about 0.02 eV from 0.77 eV in the reference solar cell to 0.79 eV for the SiN_x_:H-coated cell.

**Table 2:** Diode ideality factors (*n*), reverse saturation current densities (*J*_s_), and Schottky barrier heights (*Φ*_bi_) values of Si/PEDOT:PSS heterojunction solar cells with or without a SiN_x_:H layer

	*J*_s_ (A/cm^2^)	Diode ideality factors (*n*)	*Φ*_bi_ (eV)
W/o SiNX:H	1.08 × 10^-6^	2.59	0.77084
W/ SiNX:H	5.55 × 10^-7^	2.45	0.78794

## Conclusions

In summary, we have demonstrated that the performance of Si/PEDOT:PSS hybrid solar cells can be improved by adding a patterned passivation layer of SiN_x_:H onto the rear surface of the Si substrate. A PCE of 9 % was achieved for the SiN_x_:H-coated solar cells. Compared to the cells without rear passivation, a 0.6 % improvement in PCE was obtained. As the shrink of contact areas would increase the *R*_s_ value, further optimizations on the pattern configurations and the contact between Si and Al are needed to achieve more higher PCE for Si/PEDOT:PSS hybrid cells.

## Abbreviations

AFM, atomic force microscopy; ATR-FTIR, attenuated total reflectance Fourier transform infrared spectroscopy; *J*_sc_, photocurrent density; PCE, power conversion efficiency; PEDOT:PSS, poly(3, 4-ethylenedioxythiophene):poly(styrenesulfonate); *V*_oc_, open circuit voltage; XPS, X-ray photoelectron spectroscopy
